# Experimental caprine neosporosis: the influence of gestational stage on the outcome of infection

**DOI:** 10.1186/s13567-016-0312-6

**Published:** 2016-02-11

**Authors:** Wagnner José Nascimento Porto, Javier Regidor-Cerrillo, Pomy de Cássia Peixoto Kim, Julio Benavides, Ana Clécia dos Santos Silva, Pilar Horcajo, Andrea Alice da Fonseca Oliveira, Ignacio Ferre, Rinaldo Aparecido Mota, Luis Miguel Ortega-Mora

**Affiliations:** Campus Arapiraca-Unidade Educacional Viçosa, Universidade Federal de Alagoas, Viçosa, AL Brazil; SALUVET, Animal Health Department, Faculty of Veterinary Sciences, Complutense University of Madrid, Ciudad Universitaria s/n, 28040 Madrid, Spain; Departamento de Medicina Veterinária, Universidade Federal Rural de Pernambuco, Recife, PE Brazil; Livestock Health and Production Institute (ULE-CSIC), 24346 León, Spain

## Abstract

Here, we assessed outcome of experimental infection by *Neospora caninum* in goats intravenously inoculated with 10^6^ tachyzoites of the Nc-Spain7 isolate at 40 (G1), 90 (G2) and 120 (G3) days of gestation. Infected goats had fever between 5 and 9 days post inoculation (dpi); all were seropositive at the time of abortion/birth. Foetal death occurred in G1 from 10 to 21 dpi (*n* = 7) and in G2 from 27 to 35 dpi (*n* = 4). Goats in G2 also had seropositive stillbirth (*n* = 1) and healthy kids (*n* = 2). G3 goats (*n* = 7) had 3 seropositive and 3 seronegative weak kids, and 2 seronegative healthy kids. Parasite DNA detection in placentomes was 100% in G2, 85.7% in G3 and in G1 was detected only in placentomes from the goats with foetal losses from 17 dpi (100%). Parasites were detected in foetal/kid brain (>85.7%) and liver (≥50%) of G2 and G3, and in G1 after 17 dpi (100%). The highest parasite loads were detected in the placentomes of G1 from 17 dpi and G2, and in foetal tissues of G1 from 17 dpi and G3. Multifocal necrotic lesions were observed in the placentas of the three groups, but they were larger and more frequent in G1 and G2. Similar lesions were observed in foetal tissues, but they were more frequent in G3. These findings suggest that, as observed in cattle and sheep, the clinical consequences of *N. caninum* in pregnant goats are dependent in part on the time of gestation when animals were infected.

**Electronic supplementary material:**

The online version of this article (doi:10.1186/s13567-016-0312-6) contains supplementary material, which is available to authorized users.

## Introduction

Neosporosis is globally recognized as one of the main causes of abortion in cattle [1]. Cattle become infected during the postnatal period by ingesting oocysts shed by dogs and other canids, as well as transplacentally from the dam to the foetus after recrudescence of a chronic infection during pregnancy. After transplacental infection, foetuses may die in utero, or they may be born alive and healthy but have congenital infections or have clinical neuromuscular signs [1, 2].

Recent studies also demonstrated that neosporosis may pose a higher risk of abortion in sheep and goats than previously thought [2–7]. Caprine abortion and neonatal mortality due to *Neospora caninum* infection were first described in pygmy goats in the USA [8, 9]. Abortion and stillbirth due to neosporosis have been also reported in dairy goats in Costa Rica [10], Italy [11] and Brazil [6]. Congenital neosporosis in weak newborn goat kids manifests with neurologic signs, such as difficulty rising, ataxia, and opistothotonos, and has been observed in different farms in Brazil [12, 13]. Of note, the rate of congenital transmission of *N. caninum* in seropositive goats could be as high as the rate of transmission observed in cattle [6]. Strikingly, the clinical, epidemiology and economic importance of *N. caninum* infection in goats remain poorly investigated [2]. Serologic surveys are limited and indicate a variable antibody prevalence in goats of 2–23% according to geographical area [2]. To our knowledge, *N. caninum* has not been isolated from goats.

The pathogenesis of this disease in goats remains largely unknown as only a few experimental studies have been performed. The few studies conducted in caprine neosporosis have used pigmy goats [14]. This breed has proven to be very susceptible to experimental neosporosis because pregnant pigmy goats inoculated in early- and mid-gestation with *N. caninum* aborted infected foetuses or delivered stillborn kids [14]. The results of these studies suggest similar pathogenesis and disease outcomes as reported for cattle, in which the time point of infection during gestation plays a key role [14]. A recent study of experimental infection in sheep also demonstrated the crucial role of the stage of gestation in the course of ovine neosporosis, where infections in early- and mid-gestation caused a 100% abortion rate, whereas infections in late-gestation resulted in the birth of weak or clinically healthy infected lambs [15].

The aim of the present study was to investigate the outcome of experimental infection by *N. caninum* in goats in the early (40 day of gestation, dg), middle (90 dg) and late (120 dg) stages of pregnancy to better understand the pathogenesis of caprine neosporosis. Experimental conditions that were previously established for experimental infection in sheep were employed in this study for further comparisons between ovine and caprine neosporosis [15]. Furthermore, in the absence of broadly accepted international guidelines on models for ruminant neosporosis [16], goats were evaluated as a suitable pregnant model of neosporosis primo-infection in this study.

## Materials and methods

### Ethics committee

The procedures in this experiment were approved by the Ethics Committee for Animal Use (CEUA) of the *Universidade Federal de Alagoas* under protocol number 59/2013 and were in accordance with the current legislation of the Brazilian College of Animal Experimentation (COBEA).

### Animals and experimental design

Thirty pregnant primiparous goats included in this study came from a Boer breed flock located in the Federal University of Alagoas that was confirmed to be free of neosporosis. Goats at 12–14 months-old were selected after confirming the absence of *N. caninum* infection and other agents that could cause reproductive failure, including *Toxoplasma gondii*, *Brucella* spp., *Chlamydophila abortus*, and *Coxiella burnetii*. These animals were kept in stalls and fed twice daily with Tifton hay (*Cynodon* spp.) and a concentrate of balanced commercial rations supplemented with mineral salts and protein.

The oestrus of the animals was synchronized by using intra-vaginal sponges impregnated with progesterone for 12–14 days, after which the intra-vaginal sponges were removed and the goats were immediately intramuscularly inoculated with chorionic gonadotrophin (200 UI.). On the same day, two males negative for neosporosis were housed with females for the purpose of natural mating for 7 days. Pregnancy and foetal viability were confirmed by transabdominal ultrasonography on day 21 after mating.

The experimental design is summarized in Table 1. Of the 30 selected animals, 21 were randomly distributed into three groups (G1–G3; each *n* = 7), which were intravenously inoculated in the jugular vein with 10^6^ tachyzoites of the *N. caninum* Nc-Spain7 isolate at day 40 (G1), at day 90 (G2) and at day 120 (G3) of gestation (dg). All of the experimental infections were performed no more than 1 hour after the collection of tachyzoites from cell culture to ensure the viability of the parasite. The nine remaining animals were placed in a control group (G4; *n* = 9), and three were inoculated with PBS at each time point of tachyzoite inoculation for groups G1–G3. Three animals from G4 were culled at the average time points when abortion took place in the G1 and G2 groups or when kids were born in the G2 and G3 groups, providing a negative control for further analyses (Table 1).

**Table 1:** **Experimental design.**

Group	Number of pregnant goats	Number of foetuses/kids	Days of gestation at the time of inoculation	Inoculum (IV)
G1	7	10	40	Nc-Spain7 10^6^ tachyzoites
G2	7	8	90	Nc-Spain7 10^6^ tachyzoites
G3	7	8	120	Nc-Spain7 10^6^ tachyzoites
G4	3–3–3	3–3–3	40–90–120^a^	PBS

IV: intravenous route.

^a^In G4, 3 goats correspond to each time-point of inoculation (40, 90 and 120) and were culled at the average day of abortion of each group (i.e., 10 (*n* = 2) and 20 (*n* = 1) dpi for G1; 31 dpi for G2, *n* = 2) or parturition of kids (55 dpi for G2, *n* = 1 and 16–20 dpi for G3, *n* = 3).

### Parasites

The isolate Nc-Spain7 of *N. caninum* was maintained in a MARC-145 cell culture, as previously described [17], and the inoculum was prepared using the methodology described by Arranz-Solís et al. [15]. Briefly, tachyzoites were recovered from culture flasks when they were still largely intracellular, and at least 80% of the parasitophorous vacuoles were undisrupted. Tachyzoite number was determined by Trypan blue exclusion counting in a Neubauer chamber and parasite suspension adjusted with PBS at the required dose for inoculation. The parasites used in the inoculations did not surpass 18 passages in cell culture.

### Clinical examination and sample collection

Goats were observed daily before (−4 days post inoculation, dpi) and after inoculation and throughout the experimental period. Clinical examinations monitored foetal viability, rectal temperature and other parameters, such as appetite loss, weight loss and changes in the behaviour of the animals. Rectal temperature was recorded daily between −4 and 14 dpi and weekly from 14 dpi. Foetal viability was assessed by transabdominal ultrasonography monitoring of foetal heartbeat and movements. All goats were monitored once weekly for the 2 first weeks post-infection (wpi) and then twice weekly until detection of foetal death. When foetal death was detected, the goats were immediately euthanized by an IV overdose of sodium thiopental, followed by potassium chloride. Animals from G4 for each infected group were culled at the average time when foetal death/birth of kids was detected in groups G1, G2 and G3 (Table 1). After parturition, a clinical examination of all full-term kids was performed to determine if they had any of the following signs: weakness, neurologic signs such as difficulty rising and ataxia, and loss of the suction reflex. Three days after birth, all of the infected goats and kids were euthanized, as described above.

Blood samples were collected from the goats on the day of necropsy by jugular vein-puncture in Vacutainer tubes (Becton Dickinson and Company, Plymouth, UK) without anticoagulant and were allowed to clot. After the birth of the kids and prior to suckling colostrum, blood samples were also collected from the kids for serological analysis. Serum samples were stored at −80 °C until analysis.

Post-mortem examinations of the goats and kids were performed immediately after euthanasia, and foetuses were immediately separated from the placenta. The collection of samples for histological and molecular studies was as follows: three randomly selected placentomes were recovered from each placenta and were transversally cut into slices of 2–3 mm thickness. Placentome sections were stored in 10% formalin for histopathological examinations and −80 °C for further parasite DNA detection by PCR. When macroscopic lesions were observed in placentomes, they were selected for sampling. Iliofemoral lymph nodes were also collected from the goats for PCR. Samples of the brain and liver were collected from the foetuses and kids and stored at −80 °C for DNA extraction and PCR, as well as in 10% formalin for histological analysis. In addition, sections of the heart and semitendinosus skeletal muscle were also kept in 10% formalin for further histological processing.

### Histopathology

All samples fixed in formalin were conventionally dehydrated, embedded in paraffin and stained with haematoxylin-eosin for histological examination.

### Serological analysis (IFAT)

An indirect fluorescent antibody test (IFAT) was used to detect specific IgG anti-*Neospora* antibodies in goat sera collected at the moment of abortion/birth of kids and in precolostral sera from stillbirth to kids. A previously described IFAT analysis [18] with minor modifications was followed. Briefly, the goat sera was diluted at twofold serial dilutions, beginning at a 1:50 dilution in PBS (used as a cut-off) to the end point titre. The diluted serum in PBS was added to each well, incubated at 37 °C for 30 min and washed twice in PBS for 10 min. Then, fluorescein isothiocyanate (FITC) conjugate anti-sheep IgG (Sigma-Aldrich) at a 1:200 dilution in Evans Blue (Sigma-Aldrich) was added to the slides, incubated at 37 °C for 30 min, washed twice with PBS and once with distilled water, and then mounted with Fluoprep Solution for Immunofluorescence Technique (BioMérieux, France) for microscopic visualization. Samples were considered to be positive only if the total peripheral fluorescence was detected in more than 50% of tachyzoites in different zones of each well.

### DNA extraction and PCR for parasite detection and quantification in tissues

DNA extraction, PCR for parasite detection and quantitative real-time PCR (qPCR) for parasite quantification in tissues were performed as previously described [15]. Briefly, genomic DNA was extracted from 40 to 80 mg of maternal (uterine lymph nodes and placentome) and foetal/kid tissue samples (brain and liver) using the automated Maxwell^®^ 16 System (Promega, Wisconsin, USA). Parasite detection was carried out with 250–500 ng of sample DNA by a nested-PCR adapted to a single tube in three different DNA extractions of maternal and foetal/kid tissues using primers and conditions that were previously described [19]. Only one sample per tissue was analysed in foetuses from G1 due to the small size of recovered sample. In each round of DNA extraction and batch of PCR amplification, one sample from the uninfected control group G4 and one sample without DNA (water) were included as negative controls. The sensitivity of nested PCR was also checked in each batch of amplification, including samples of genomic DNA equivalent to 10, 1 and 0.1 Nc-Spain7 tachyzoites in 100 ng of goat DNA.

Positive samples by nested-PCR from the placental, brain and liver tissues were adjusted to 20 ng/μL and the parasite burden was quantified using qPCR, as previously described [15]. Briefly, primer pairs of the *N. caninum* Nc-5 sequence and β-actin gene were used to quantify parasite and host DNA by interpolating the sample Ct values on two standard curves as follows: (1) equivalent to 5 × 10^5^ to 5 × 10^−1^ tachyzoites with 10-fold serial dilutions in a solution of genomic goat DNA and (2) a curve of 320, 160, 80, 40, 20, 10, and 5 ng of genomic goat DNA. The quantity of parasites in the tissue samples (parasite burden) was expressed as parasite number/mg goat tissue. Standard curves for *N. caninum* and goat DNA exhibited a mean slope of −3.79 and −3.47, respectively, as well as an R^2^ > 0.99.

### Statistical analysis

Rectal temperature values for each experimental group throughout the time course were analysed using a one-way ANOVA (G1) and one-way ANOVA of repeated measures (G2, G3 and G4). When statistically significant differences were found, Tukey’s Multiple Range test was applied to examine all possible pair-wise comparisons at each sampling time. Differences in the PCR detection of parasite DNA were assessed using the *χ*^2^ or Fishers exact F-test. Differences in parasite burden were analysed using the non-parametric Kruskal–Wallis test, followed by Dunn’s test for comparisons between groups and the Mann–Whitney test. The statistical significance for all tests was set at *P* < 0.05. All statistical analyses were performed using GraphPad Prism 5.0 software.

## Results

### Clinical findings

**Figure 1:**
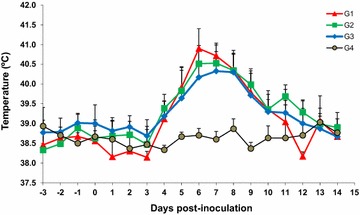
**Rectal temperatures.** Mean rectal temperatures (+SD) recorded from goats inoculated with 10^6^ Nc-Spain7 tachyzoites at day 40 of gestation (G1), at day 90 of gestation (G2), at day 120 of gestation (G3) and with PBS control group (G4) for 14 days post-inoculation (see legend).

The goats in G1, G2 and G3 had a fever (rectal temperature ≥39.5 °C) from 5 to 9 dpi, with a maximum mean temperature at 6–7 dpi (ANOVA, *P* < 0.0001) (Figure 1). Fevers persisted in 2 of 7 animals in G2 until 13 dpi and 1 of 7 animals in G3 until 11 dpi. Fever was accompanied in the infected animals by apathy, decreased appetite and bristly hair. Fever was not detected in the infected animals from 14 dpi or in the control animals (G4) throughout the experiment.

Foetal death was detected by transabdominal ultrasonography in all goats (100%) from group G1. Four goats from G1 had foetal death during the second wpi, between 10 and 11 dpi, and the other three goats in this group exhibited foetal death during the third wpi, between 17 and 21 dpi (Table 2; Additional file 1). Revision of rectal temperatures in the G1 group showed that goats aborting during the second wpi had earlier fever, at 4 dpi, and above 40 °C from 5 to 9 dpi, whereas in goats aborting during the third wpi, fever appeared at 5 dpi and exceeded 40 °C only from 6 to 8 dpi (Additional file 1). Nevertheless, a significant increase was found only at 4–5 dpi in G1 aborted during the second wpi vs G1 aborted during the third wpi (*P* < 0.05).

**Table 2:** **Clinical outcome, histology findings and PCR detection and quantification of** ***N. caninum*** **in the placenta and foetal liver and brain.**

Group	Foetal death (dpi)	Birth (dpi)	Placenta		Liver		Brain	
			Histology^b^	PCR^c^	Histology^b^	PCR^c^	Histology^b^	PCR^c^
G1^a^	10–11	–	–	1/4 (0.001)	–	0/4 (0)	–	0/4 (0)
	17–21	–	++	3/3 (30 802)	++	3/3 (9828)	+	3/3 (10 213)
G2	27–35	53–55^d^	++	7/7 (31 455)	+	3/6 (0.001)	++	7/8 (11.5)
G3	–	12–22^e^	+	6/7 (732.5)	++	7/8 (345.4)	++	7/8 (28.27)

dpi: days post infection when abortion occurred.

^a^The G1 group was divided due to the differences found (histology, detection and burdens), depending on whether the animals aborted during the second or third wpi.

^b^Histopathological lesion severity: none detected (–), consistent with (+), and characteristic of (++) *N. caninum* infection.

^c^Fractions represent the number of positive animals/total number of animals assessed by nested-ITS1 PCR, and figures within brackets represent the median values of parasite burden (tachyzoites/mg tissue).

^d^Three goats kidded. One goat gave birth to a stillbirth at 143 days of gestation, and two gave birth to healthy kids at 143 and 145 days of gestation.

^e^All goats kidded between 132 and 142 days of gestation giving birth to 8 kids, of which 6 showed clinical signs of weakness.

In G2, the loss of foetal viability occurred later in four goats, between 27 and 35 dpi, whereas the other three infected goats of this group gave birth to a stillborn kid at 143 days of gestation (53 dpi) and two healthy kids at 143 and 145 dg (53–55 dpi) (Table 2; Additional file 1).

All goats from G3 gave birth to a total of eight kids between 132 and 142 dg (12–22 dpi), of which six displayed weakness and were unable to rise and suckle from their mother (Table 2; Additional file 1). Excluding abortion and stillbirth, additional clinical signs of illness associated with neosporosis were not observed in the inoculated goats.

Foetal death was not observed in the G4 control group prior to programmed culling at 10 and 20 dpi (corresponding controls for G1), at 31 and 55 dpi (corresponding controls for G2) and at 16–20 dpi (136–140 dg) (corresponding controls for G3).

### *Neospora*-specific IgG responses

*Neospora*-specific IgG responses were analysed by IFAT in goat sera (Additional file 1). All goats from G1, G2 and G3 showed a specific *N. caninum* IgG response in sera. The goats from G1 that aborted during the second wpi showed the lowest titres from 1:50 to 1:400, indicating recent seroconversion, which increased in G1 goats that aborted later, from 1:800 to 1:3200. Titres in goat from G2 varied from 1:400 to 1:1600, whereas those in the goats from G3 ranged from 1:200 to 1:800.

Seropositive precolostral titres were also detected in the stillborn kid and two kids born from G2 goats, ranging from 1:1600 to 1:3200. Most kids born from goats from G3 were seronegative, and seroconversion was detected only in three kids born at 18 and 22 dpi with titres of 1:100 and 1:200 (Additional file 1).

Specific IgG responses against parasite antigen were not detected in kids from the control group (G4).

### Anatomical and histopathological findings

At the time of culling, most of the infected dams in which foetal death was detected by transabdominal ultrasonography (all from G1 to 57% from G2) showed detachment of the placental surface from the uterus. There was abundant turbid and yellowish liquid in the uterus, and the surface of the uterine caruncles was congestive and showed fibrin deposition. All foetuses from these dams were dead, and the foetal viscera were oedematous, congestive and friable, suggesting an advanced degree of autolysis.

**Figure 2:**
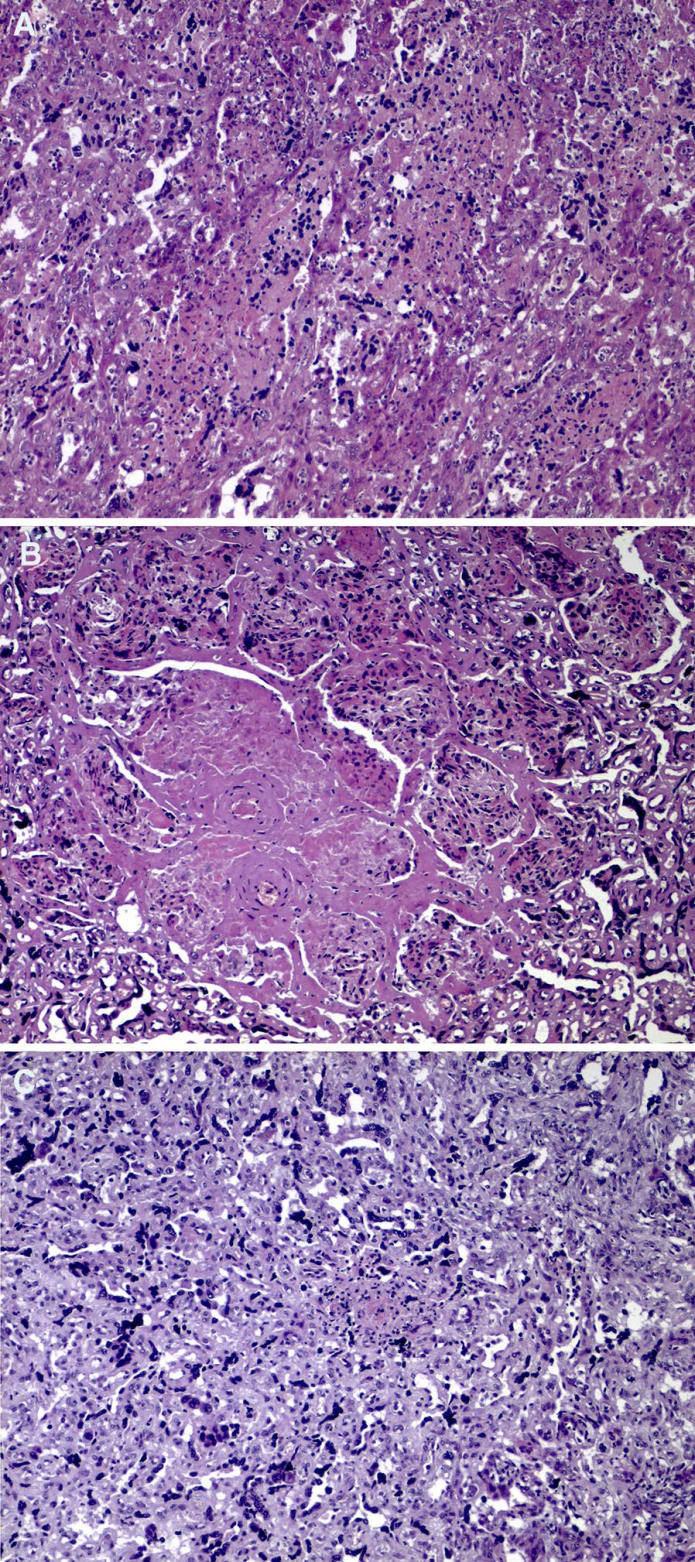
**Microscopic lesions found in the placenta.** **A** G1. Multifocal necrotic placentitis with very scant infiltration of inflammatory cells. **B** G2. Multifocal to coalescent severe necrotic placentitis. Note that inflammatory cells are found at the periphery of the necrotic foci. **C** G3. Isolated foci of necrosis, where the infiltration of inflammatory cells is more evident than in the placentas from G2 and G1. All pictures were taken at the same magnification. Bar 200 μm.

Microscopically, in the placentas from G1 goats aborted during the third wpi and one goat aborted during the second wpi (identified by number 115), as well as those from G2, there was mild multifocal non purulent necrotic placentitis characterized by randomly distributed foci of necrosis in the interdigitated area of the placentome (Figure 2). Lesions were similar between the two groups, both in the number and size of foci. The other placentas from G1 goats aborted in the second wpi showed patchy congestion of the interdigitated area. The placentas from G3 showed very mild lesions, usually only one or two small necrotic foci were found in the placentomes (Figure 2).

Histological lesions in the liver and brain were characterized by the presence of multiple foci of coagulative necrosis that were randomly distributed in the parenchyma. Only one foetus from G1 goats aborted in the second wpi showed lesions, which were primarily necrotic. In the rest foetuses aborted from this group there were no significant histological changes. Nevertheless, the portion of brain sample from G1 examined was substantially reduced due to foetal autolysis. All foetuses from G2 to G3 had lesions in these locations and they were characterized by a more conspicuous infiltration of inflammatory cells, especially in kids from G3.

### Parasite distribution and burden in placental, foetal and maternal tissues

#### Maternal and placental tissues

*Neospora caninum* DNA was detected in uterine lymph nodes in a variable number of infected goats, depending on the group. Parasite DNA was detected in 5 out of 7 goats from groups G2 and G3 and in 3 out of 7 in G1. Significant differences in the frequency of detection were found only between G2, which had the highest detection (13/21), and G1, which had the lowest detection (4/21) (*P* < 0.05) (Additional file 1).

**Figure 3:**
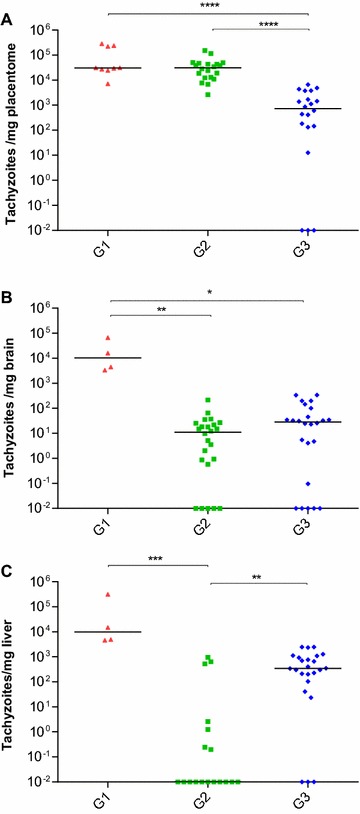
**Dot-plot graph of** ***N. caninum*** **burdens.** Parasite burdens were quantified by real-time PCR in the placenta (**A**), foetal brain (**B**) and liver (**C**) from animals inoculated with 10^6^ Nc-Spain7 tachyzoites at days 40—G1, 90—G2 and 120—G3 of gestation. Data from G1 goats that aborted during the second wpi are excluded because they were mainly negative according to PCR detection (the only positive sample had a burden close to the detection limit by real-time PCR, 0.1). Each dot represents individual values of parasite burden (number of parasites per mg of host tissue), and medians are represented as horizontal lines. Taking into account that the *N. caninum* detection limit by real-time PCR is 0.1 parasites, negative samples (0 parasites) were represented on the log scale as <0.1 (i.e., 10^−2^). (*) indicates *P* < 0.05, (**) *P* < 0.005, (***) *P* < 0.001 and (****) *P* < 0.0001.

Parasite DNA was detected in placentomes from four goats in G1, from all goats aborted during the third wpi (3/3) and from one goat that aborted during the second wpi (identified by number 115) (1/4). In G2, parasite DNA was detected in all animals (7/7) and almost all goats from G3 (6/7). The only goat from G3 in which parasite was not detected gave birth to a healthy kid at 12 dpi (132 dg) (Additional file 1). With regards to the frequencies of detection, parasite DNA was widely detected in G2 (21/21), G3 (18/21) and in goats aborted during the third wpi (9/9) in G1; the goats aborted during the second wpi in G1 showed a significantly lower frequency of detection (1/12) than the goats in the other infected groups (*P* < 0.0001). The highest mean parasite burden, which was measured as the number of tachyzoites per mg of tissue, was found in placentomes from the G1 animals that aborted during the third wpi and in G2 compared with the animals from G3 (*P* ≤ 0.001) (Figure 3A).

No parasite was detected in the lymph nodes and placental tissues from the control goats (G4).

#### Foetal tissues

Parasite DNA was not detected in aborted foetuses during the second wpi from G1, whereas all aborted foetuses during third wpi were PCR-positive, with the highest frequency of detection in brain and liver samples (100% of samples). Parasite DNA was also detected in brain and/or liver tissues from all foetuses and kids of G2, although it was not detected in tissues from the stillborn kid delivered at 53 dpi (143 dg; goat number 70) (Table 2; Additional file 1). Similarly, parasite DNA was amplified in the majority of kids from G3. PCR detection failed in tissues from the first kid born at 12 dpi (132 dg; number 67) and was poorly detected in the brain from the kid born at 15 dpi (135 dg; number 66) who remained healthy after birth (Table 2; Additional file 1). The frequency of detection in brain samples was high and very similar between G2 and G3 (79%, 19/24), but in liver samples, the frequency of detection in animals from G2 was significantly lower (39%, 7/18) than in G3 (88%, 21/24) (*P* < 0.005).

The highest parasite burden in the brain was found in foetuses from G1 that were aborted during the third wpi, which had significantly higher burdens than the other two groups G2 (*P* < 0.005) and G3 (*P* < 0.05) (Figure 3B). The parasite burdens in the brains of the aborted foetuses from G2 were significantly higher than those in the brains of delivered kids (*P* < 0.05) of the same group. In the liver, significant differences were found among the groups. The highest burdens were found in G1 aborted during the third wpi and G3 compared with G2 (*P* < 0.005) (Figure 3C). Differences were not detected regarding the parasite burden in the livers between the aborted foetuses and the kids from G2. However, the parasite burden in the livers of kids from G3 that showed clinical signs were significantly higher than the burdens detected in healthy kids (*P* < 0.001).

As expected, all foetal samples from G4 were negative.

## Discussion

Although *N. caninum* is currently recognized as one of the most important infectious causes of abortion in cattle worldwide [1, 2], its epidemiological, clinical and economic relevance for reproductive failure in small ruminants have remained poorly understood. Recently, an exhaustive study was carried out in sheep to characterize the clinical consequences, dynamics of infection and lesion development after infection in the early (40 dg), middle (90 dg) and late (120 dg) stages of gestation. The study demonstrated the influence of the stage of gestation on the course of infection and the strong similarities with natural ovine neosporosis [15]. Similar results have been previously shown in bovine neosporosis [20].

The aim of this study was to reproduce *N. caninum* infections in goats under analogous experimental conditions used in sheep for proper comparison with experimental neosporosis in sheep [15] and cattle [19, 21]. The crucial role played by time of pregnancy for infection in the outcome of neosporosis was also demonstrated in goats. The clinical outcome of infection resulted in foetal death in all goats inoculated during the first term of gestation (G1). Foetal death was also recorded in goats inoculated during the second term of gestation (G2), although infection at day 90 also caused one stillborn kid and the birth of healthy but congenitally infected kids. In contrast, infection during the last term of gestation (G3) resulted in the birth of full-term congenitally infected kids, although most of them showed weakness and neurologic signs that could compromise their future viability.

Infection during the first period of gestation (G1) had similar consequences (100% of foetal death) to previous experimental studies in cattle, where foetal death occurred approximately 4–5 wpi [19, 22, 23], and in sheep, where abortion was observed during the third wpi [15, 24]. The abortions occurred in the current study during 17–21 dpi were similar to those reported in sheep during the same period [15]. They were characterized by vast parasite dissemination in the placenta and foetal tissues. They achieved high parasite burdens but mild lesions in the placenta [15, 24]. Although a high parasite burden has also been described in bovine neosporosis at this time of gestation, in this species, it is related to extensive necrosis and inflammation in the placenta [19, 23, 25]. These results suggest differences between cattle and small ruminants in the dynamics of *N. caninum* infection during early pregnancy, as denoted by the apparently limited time of parasite proliferation in the placenta of small ruminants, which limits the development of lesions. This fact may be caused by a shorter time of abortion occurrence (21 days in small ruminants vs 28 in cattle). However, the scarcity of lesions in the placenta does not impair its timely crossing of the placenta by the parasite, its rapid multiplication, or its vast spreading in foetal tissues. A previous experimental infection in pigmy goats also showed a 100% abortion rate and reported mild, if any, lesions in the placenta [14]. These considerations support that abortion for early pregnancy in small ruminants may be a direct consequence of damage caused by the multiplication of the parasite in tissues of the foetus that is immunologically immature [15], whereas placental lesions could also contribute to the occurrence of abortion in cattle [19, 23].

The abortions that occurred in the G1 group during the second wpi deserve special attention. They were characterized by the detection of parasite DNA, which was limited to only scant placentomes from one dam; none was found in foetal samples. These findings indicate a recent spread of the parasite to the placenta. Moreover, there were no lesions in placentomes. Nevertheless, a specific IgG response was detected by IFAT in all infected goats. Interestingly, similar findings have been described for abortions occurring during the acute phase of experimental toxoplasmosis in sheep [26, 27] and in goats [28]. Likewise, limited or absent parasite dissemination was revealed in the placenta and foetal tissues, respectively. However, the infected sheep in those studies remained seronegative for *T. gondii* during the acute phase [26, 27], and vascular lesions were found in the placenta [27]. It has been proposed that these early abortions may be caused by pyrexia before tachyzoites reach placenta [29]. Analysis of the rectal temperatures in goats that aborted in the second week post infection had a prompter fever onset and a longer peak than those that aborted during the third week. This finding may suggest that this peak of hyperthermia could be related to, or at least participate in, the triggering of abortions and that this mechanism was different from that related to the abortions occurring one week later. Additional studies would be necessary to establish the particular pathogenesis of abortion during the acute phase of neosporosis in goats. Furthermore, goats may be more sensible to *N. caninum* infection since foetal death during the acute phase of the disease have not been described in sheep or cattle.

Infection during mid-gestation (G2) caused more heterogeneous results. Abortion occurred at 4–5 wpi in four out of seven goats; there was one stillborn kid, and two goats delivered congenitally infected healthy kids. Similar findings were reported in previous experimental infections in goats [14]. Abortions during the second and early-third terms, as well as stillbirths, have been reported in naturally infected goats [6, 8, 10, 11]. In comparison with sheep, under the same experimental conditions, goats appear to be less susceptible to Nc-Spain7 infection during this period of gestation because foetal death was recorded in all Nc-Spain7 infected sheep during mid-gestation, although it occurred later, at 5–7 wpi [15]. Abortion was also the main consequence of subcutaneous administration of 10^6^ Nc-1 [24] and occurred in 100% of pregnant sheep after inoculation of 10^6^ of a mix of NcNZ1, NcNZ2 and NcNZ3 tachyzoites at 90 dg [30]. The results in goats were more similar to those observed in cattle because abortions and births have been described in both species after infections during the second term of gestation, although the results heavily depend on the experimental design of the studies. *N. caninum* infection in cattle during the second term of gestation, usually after 120 dg, commonly causes the birth of full-term live but congenitally infected calves [31–33]. However, abortion has also been reported after inoculation of *N. caninum* at 110 dg [33], and 3 out of 6 cattle infected at 110 dg with 10^7^ tachyzoites of Nc-Spain7 suffered foetal death at 2–3 wpi [21]. In addition, the second term of gestation is recognized as the period when most of the abortions occur in natural bovine neosporosis [1, 34]. Interestingly, similar disseminations, parasite burdens and lesion severities were observed in placentas from goats of G1 that aborted during the third wpi and goats of G2 that aborted foetuses and full-term healthy kids born three weeks later, suggesting a certain control of parasite infection in the placenta from mid-gestation that is capable of limiting parasite proliferation but is insufficient to eliminate it from this location. In this sense, it has been described that there could be a shift of the immune system of ruminants from mid- to late-gestation towards a T-helper 2-type response at the materno-foetal interface to maintain foetal viability that favours parasite multiplication and transmission to the foetus [35]. Moreover, although the severity of the lesions in both the placentas and the foetuses was similar to that found in G1 goats that aborted during the third wpi, parasite detection in foetal tissues was lower in G2 foetus/kids, particularly in the liver. On the other hand, the kids born in G2 showed the highest *N. caninum* IgG response according to IFAT titres, and the foetal lesions showed a more conspicuous inflammatory component. The maturation of the foetal immune system during the second term of gestation is most likely responsible of these findings. At this stage of gestation, some control of the infection is possible in the foetus, as shown by comparing the foetuses that survived throughout gestation with those that died. The former showed the lowest parasite burden in the brain and milder lesions in the foetal viscera, despite broad dissemination of the parasite and pathological changes in the placenta, suggesting that there was some control of parasite dissemination and proliferation in foetal tissues, which could have determined their survival until delivery. Surprisingly, the stillborn kid in this group had no *N. caninum* DNA, although it exhibited a high IFAT titre and characteristic histological lesions in the placenta, suggesting previous contact with the parasite in the uterus.

Infection during the final term of gestation (G3) resulted in the birth of congenitally infected premature kids (between 132 and 142 dg), most of which showed clear weakness and clinical nervous signs associated with infection; these animals would not have survived the first weeks of life had they not been euthanized. The consequences of infection at 120 dg resemble those described in sheep infected with the same isolate at late-gestation [15]. A previous experimental infection of goats at late-gestation also resulted in birth of weak kids, although congenital infection in this case could not be proven [14]. The birth of weak kids showing neurologic signs has also been described in naturally infected goats [12, 13]. The results from these studies suggest that *N. caninum* infection of goats and sheep in late-gestation may have more severe consequences than infection of cattle at a similar period [22, 23, 36]. Cattle infected at this period deliver healthy but congenitally infected calves, which is likely due to a longer period time of gestation from inoculation to delivery, better control of infection by the maternal and foetal immune system, and healing of tissue damage caused by the parasite during the last weeks of gestation, all of which consequently lead to the delivery of a healthy calf [36]. Interestingly, during this period of gestation, parasite spreading in the placentomes of small ruminants apparently occurs earlier, from week 2 post-inoculation, and is more widespread, occurring in all placentomes. This is different than in cattle, where spreading only occurs in a few placentomes per animal, and parasite DNA is present only from week 4 post-inoculation [36]. Although less intense than in the second term of gestation, the early and broad colonization of the placenta occurring after infection in the third term may trigger the premature delivery of kids and lambs. More interestingly, parasite transmission/proliferation may be more efficient, or foetal immune control may be less efficient, at this stage of gestation in goats than in sheep because apparently higher parasite frequencies and burdens were detected in the brain and liver of kids compared with lambs infected under similar circumstances [15]. In addition, and despite widely disseminated infection, the IgG response in kids was absent in the majority of cases or was very low at the best, suggesting impaired development of the humoral immune response at that time. Similar to the description in ovine experimental neosporosis, the frequencies of detection and parasite burdens found in kids showing clinical signs were significantly higher than in healthy kids. It is tempting to hypothesize that in the later, parasite transmission/proliferation may have been better controlled by the foetal immune response, resulting in clinically non-affected kids.

Based on the results obtained in the present study, it is possible to conclude that the consequences of neosporosis in pregnant goats mainly depends on the time of gestation when infection occurs, which is a finding that is similar to the results described for sheep and cattle. Experimental infection of goats during the first term of gestation resulted in abortion, which is a finding that is similar to those described in sheep and cattle, mainly, by parasite proliferation and damage in foetal tissues in small ruminants, and likely with contribution of placental damage in cattle. The results of infection during the second term of gestation were highly similar to those in natural and experimental infections in cattle and sheep when immunocompetence and control of *N. caninum* infection in the foetus begin, and damage to the placenta could be related to the outcome of infection. Infections during the third term of gestation are more similar in sheep and goats, which birth premature and weak kids, compared with cattle, which birth healthy congenitally infected calves, which is likely due to control of infection and damage in cattle foetuses during the final weeks of pregnancy. These findings warrant further characterization of caprine neosporosis as a disease of increasing importance in goats. Despite of divergences with bovine, a neosporosis model in goats based on tachyzoite infection during the second term of gestation seems suitable for evaluation of vaccine and drug candidates, due to the similar clinical characteristics and outcomes of infection. Nevertheless, further studies would be desirable for improvement and standardizing of *N. caninum* infection model in goats [16].

## Additional files

10.1186/s13567-016-0312-6 **Summary of individual results.** Individual clinical signs, parasite DNA detection and serological results in dams and foetuses/kids.
10.1186/s13567-016-0312-6 **Mean rectal temperatures in G1.** Mean rectal temperatures (+SD) recorded from goats inoculated with 10^6^ Nc-Spain7 tachyzoites at day 40 of gestation that suffered foetal death during the second wpi (G1A) or during the third wpi (G1B) (see legend).

## References

[1] Dubey JP, Schares G, Ortega-Mora LM (2007). Epidemiology and control of neosporosis and *Neospora caninum*. Clin Microbiol Rev.

[2] Dubey JP, Schares G (2011). Neosporosis in animals—the last five years. Vet Parasitol.

[3] Hassig M, Sager H, Reitt K, Ziegler D, Strabel D, Gottstein B (2003). *Neospora caninum* in sheep: a herd case report. Vet Parasitol.

[4] Howe L, Collett MG, Pattison RS, Marshall J, West DM, Pomroy WE (2012). Potential involvement of *Neospora caninum* in naturally occurring ovine abortions in New Zealand. Vet Parasitol.

[5] Moreno B, Collantes-Fernández E, Villa A, Navarro A, Regidor-Cerrillo J, Ortega-Mora LM (2012). Occurrence of *Neospora caninum* and *Toxoplasma gondii* infections in ovine and caprine abortions. Vet Parasitol.

[6] Mesquita LP, Nogueira CI, Costa RC, Orlando DR, Bruhn FRP, Lopes PFR, Nakagaki KYR, Peconick AP, Seixas JN, Júnior PSB, Raymundo DL, Varaschin MS (2013). Antibody kinetics in goats and conceptuses naturally infected with *Neospora caninum*. Vet Parasitol.

[7] González-Warleta M, Castro-Hermida JA, Regidor-Cerrillo J, Benavides J, Álvarez-García G, Fuertes M, Ortega-Mora LM, Mezo M (2014). *Neospora caninum* infection as a cause of reproductive failure in a sheep flock. Vet Res.

[8] Barr BC, Anderson ML, Woods LW, Dubey JP, Conrad PA (1992). *Neospora*-like protozoal infections associated with abortion in goats. J Vet Diagn Invest.

[9] Dubey JP, Acland HM, Hamir AN (1992). *Neospora caninum* (Apicomplexa) in a stillborn goat. J Parasitol.

[10] Dubey JP, Morales JA, Villalobos P, Lindsay DS, Blagburn BL, Topper MJ (1996). Neosporosis-associated abortion in a dairy goat. J Am Vet Med Assoc.

[11] Eleni C, Crotti S, Manuali E, Costarelli S, Filippini G, Moscati L, Magnino S (2004). Detection of *Neospora caninum* in an aborted goat foetus. Vet Parasitol.

[12] Corbellini LG, Colodel EM, Driemeier D (2001). Granulomatous encephalitis in a neurologically impaired goat kid associated with degeneration of *Neospora caninum* tissue cysts. J Vet Diagn Invest.

[13] Varaschin MS, Hirsch C, Wouters F, Nakagaki KY, Guimaraes AM, Santos DS, Bezerra PS, Costa RC, Peconick AP, Langohr IM (2012). Congenital neosporosis in goats from the State of Minas Gerais, Brazil. Korean J Parasitol.

[14] Lindsay DS, Rippey NS, Powe TA, Sartin EA, Dubey JP, Blagburn BL (1995). Abortions, fetal death, and stillbirths in pregnant pygmy goats inoculated with tachyzoites of *Neospora caninum*. Am J Vet Res.

[15] Arranz-Solís D, Benavides J, Regidor-Cerrillo J, Fuertes M, Ferre I, del Ferreras M, Collantes-Fernández E, Hemphill A, Perez V, Ortega-Mora LM (2015). Influence of the gestational stage on the clinical course, lesional development and parasite distribution in experimental ovine neosporosis. Vet Res.

[16] Benavides J, Collantes-Fernández E, Ferre I, Perez V, Campero C, Mota R, Innes E, Ortega-Mora LM (2014). Experimental ruminant models for bovine neosporosis: what is known and what is needed. Parasitology.

[17] Regidor-Cerrillo J, Gómez-Bautista M, Pereira-Bueno J, Adúriz G, Navarro-Lozano V, Risco-Castillo V, Fernández-García A, Pedraza-Díaz S, Ortega-Mora LM (2008). Isolation and genetic characterization of *Neospora caninum* from asymptomatic calves in Spain. Parasitology.

[18] Álvarez-García G, Collantes-Fernández E, Costas E, Rebordosa X, Ortega-Mora LM (2003). Influence of age and purpose for testing on the cut-off selection of serological methods in bovine neosporosis. Vet Res.

[19] Regidor-Cerrillo J, Arranz-Solís D, Benavides J, Gomez-Bautista M, Castro-Hermida JA, Mezo M, Perez V, Ortega-Mora LM, Gonzalez-Warleta M (2014). *Neospora caninum* infection during early pregnancy in cattle: how the isolate influences infection dynamics, clinical outcome and peripheral and local immune responses. Vet Res.

[20] Rosbottom A, Gibney EH, Guy CS, Kipar A, Smith RF, Kaiser P, Trees AJ, Williams DJ (2008). Upregulation of cytokines is detected in the placentas of cattle infected with *Neospora caninum* and is more marked early in gestation when fetal death is observed. Infect Immun.

[21] Darwich L, Li Y, Serrano B, Mur R, García-Isperto I, Regidor-Cerrillo J, Cabezón O, Ortega-Mora LM, López-Gatius F, López-Gatius F, Almería S (2015). INFγ production in *Neospora caninum* experimentally infected dams at 110 days of gestation and in their fetuses.

[22] Williams DJ, Guy CS, McGarry JW, Guy F, Tasker L, Smith RF, MacEachern K, Cripps PJ, Kelly DF, Trees AJ (2000). *Neospora caninum*-associated abortion in cattle: the time of experimentally-induced parasitaemia during gestation determines foetal survival. Parasitology.

[23] Gibney EH, Kipar A, Rosbottom A, Guy CS, Smith RF, Hetzel U, Trees AJ, Williams DJ (2008). The extent of parasite-associated necrosis in the placenta and foetal tissues of cattle following *Neospora caninum* infection in early and late gestation correlates with foetal death. Int J Parasitol.

[24] Buxton D, Maley SW, Wright S, Thomson KM, Rae AG, Innes EA (1998). The pathogenesis of experimental neosporosis in pregnant sheep. J Comp Pathol.

[25] Macaldowie C, Maley SW, Wright S, Bartley P, Esteban-Redondo I, Buxton D, Innes EA (2004). Placental pathology associated with fetal death in cattle inoculated with *Neospora caninum* by two different routes in early pregnancy. J Comp Pathol.

[26] Owen MR, Clarkson MJ, Trees AJ (1998). Acute phase *toxoplasma* abortions in sheep. Vet Rec.

[27] Castano P, Fuertes M, Ferre I, Fernández M, Mdel Ferreras C, Moreno-Gonzalo J, Gonzalez-Lanza C, Katzer F, Regidor-Cerrillo J, Ortega-Mora LM, Perez V, Benavides J (2014). Placental thrombosis in acute phase abortions during experimental *Toxoplasma gondii* infection in sheep. Vet Res.

[28] Dubey JP (1981). *Toxoplasma*-induced abortion in dairy goats. J Am Vet Med Assoc.

[29] Edwards MJ, Walsh DA, Li Z (1997). Hyperthermia, teratogenesis and the heat shock response in mammalian embryos in culture. Int J Dev Biol.

[30] Weston JF, Howe L, Collett MG, Pattison RS, Williamson NB, West DM, Pomroy WE, Syed-Hussain SS, Morris ST, Kenyon PR (2009). Dose-titration challenge of young pregnant sheep with *Neospora caninum* tachyzoites. Vet Parasitol.

[31] Barr BC, Rowe JD, Sverlow KW, BonDurant RH, Ardans AA, Oliver MN, Conrad PA (1994). Experimental reproduction of bovine fetal *Neospora* infection and death with a bovine *Neospora* isolate. J Vet Diagn Invest.

[32] Innes EA, Wright SE, Maley S, Rae A, Schock A, Kirvar E, Bartley P, Hamilton C, Carey IM, Buxton D (2001). Protection against vertical transmission in bovine neosporosis. Int J Parasitol.

[33] Almería S, De Marez T, Dawson H, Araujo R, Dubey JP, Gasbarre LC (2003). Cytokine gene expression in dams and foetuses after experimental *Neospora caninum* infection of heifers at 110 days of gestation. Parasite Immunol.

[34] Dubey JP, Buxton D, Wouda W (2006). Pathogenesis of bovine neosporosis. J Comp Pathol.

[35] Innes EA, Wright S, Bartley P, Maley S, Macaldowie C, Esteban-Redondo I, Buxton D (2005). The host-parasite relationship in bovine neosporosis. Vet Immunol Immunopathol.

[36] Benavides J, Katzer F, Maley SW, Bartley PM, Canton G, Palarea-Albaladejo J, Purslow CA, Pang Y, Rocchi MS, Chianini F, Buxton D, Innes EA (2012). High rate of transplacental infection and transmission of *Neospora caninum* following experimental challenge of cattle at day 210 of gestation. Vet Res.

