# Supplementary material for: Experimental caprine neosporosis: the influence of gestational stage on the outcome of infection
Source: Vet Res. 2016 Feb 11;47:29. doi: 10.1186/s13567-016-0312-6 (PMC4750177; doi:10.1186/s13567-016-0312-6)
Supplement: Supplementary file 1 — 10.1186/s13567-016-0312-6 Summary of individual results. Individual clinical signs, parasite DNA detection and serological results in dams and foetuses/kids. [file 13567_2016_312_MOESM1_ESM.docx]

**Additional file 1 Individual frequency of parasite detection by PCR in foetuses/kids at the time of necropsy and serological findings.**

| **Group** | **Goat ref.** | **Time of necropsy^a^ (dpi)** | **Time of pregnancy^b^ (dg)** | **PCR^c^** | | **Serology**  **(IFAT)** | **Foetus/ Kid ref.** | **PCR^c^** | | **Serology**  **(IFAT)** |
| --- | --- | --- | --- | --- | --- | --- | --- | --- | --- | --- |
|  |  |  |  | **Pla.** | **LN** |  |  | **Brain** | **Liver** |  |
| **G1**  **(day 40)** | **109** | 10 | 50 | **-** | **+** | 1:400 | **F109** | - | - | NA |
|  | **115** | 10 | 50 | **+** | **+** | 1:100 | **F115** | - | - | NA |
|  | **111** | 11 | 51 | **-** | **-** | 1:50 | **F111** | - | - | NA |
|  | **149** | 11 | 51 | **-** | **-** | 1:400 | **F149-1** | - | - | NA |
|  |  |  |  |  |  |  | **F149-2** | NA | NA | NA |
|  |  |  |  |  |  |  | **F149-3** | NA | NA | NA |
|  | **139** | 17 | 57 | **+++** | **-** | 1:800 | **F139** | + | + | NA |
|  | **107** | 21 | 61 | **+++** | **++** | 1:3200 | **F107** | + | + | NA |
|  | **133** | 21 | 61 | **+++** | **-** | 1:800 | **F133-1** | + | + | NA |
|  |  |  |  |  |  |  | **F133-2** | + | + | NA |
| **G2**  **(day 90)** | **116** | 27 | 117 | **+++** | **+++** | 1:800 | **F116** | +++ | NA | NA |
|  | **108** | 29 | 119 | **+++** | **-** | 1:800 | **F108** | +++ | NA | NA |
|  | **112** | 29 | 119 | **+++** | **+++** | 1:800 | **F112** | +++ | - | NA |
|  | **65** | 35 | 125 | **+++** | **+** | 1:1600 | **F65-1** | +++ | +++ | NA |
|  |  |  |  |  |  |  | **F65-2** | +++ | + | NA |
|  | **120** | 53 | 143 | **+++** | **+++** | 1:400 | **K120** | + | +++ | 1:3200 |
|  | **70** | 53 | 143 | **+++** | **-** | 1:800 | **F70*** | - | - | 1:6400 |
|  | **64** | 55 | 145 | **+++** | **+++** | 1:1600 | **K64** | +++ | - | 1:6400 |
| **G3**  **(day 120)** | **67** | 12 | 132 | **-** | **+** | 1:400 | **K67** | - | - | - |
|  | **126** | 14 | 134 | **+++** | NA | 1:200 | **K126‡** | +++ | +++ | - |
|  | **66** | 15 | 135 | **+++** | **++** | 1:200 | **K66** | + | +++ | - |
|  | **117** | 16 | 136 | **+++** | **+** | 1:400 | **K117‡** | +++ | +++ | - |
|  | **103** | 18 | 138 | **+++** | **++** | 1:200 | **K103-1‡** | +++ | +++ | 1:200 |
|  |  |  |  |  |  |  | **K103-2‡** | +++ | +++ | 1:100 |
|  | **123** | 21 | 141 | **+++** | **-** | 1:800 | **K123‡** | +++ | +++ | - |
|  | **141** | 22 | 142 | **+++** | **-** | 1:800 | **K141‡** | +++ | +++ | 1:200 |

^a^ necropsies were carried out when foetal dead was detected or after parturition.

^b^ Time of pregnancy or day of parturition.

^c^ Frequency of parasite detection by PCR in maternal and foetal tissues: (-) not detected; (+) detection in 1 out of 3 checked samples; (++) detection in 2 out of 3 checked samples; (+++); detection in all 3 samples. Note that only a sample of foetal brain and liver from G1 group was available for PCR analysis.

^*^ stillbirth

‡ kid prematurely born showing weakness and unable to rise and suckle from their mother.

dpi: days post-infection; dg: days of gestation; Pla.: placentome; LN: lymph nodes (iliofemoral); F: foetus; K: kid; NA: not available.
