# Supplementary figures and images for: Experimental caprine neosporosis: the influence of gestational stage on the outcome of infection
Source: Vet Res. 2016 Feb 11;47:29. doi: 10.1186/s13567-016-0312-6 (PMC4750177; doi:10.1186/s13567-016-0312-6)

## Slide 1
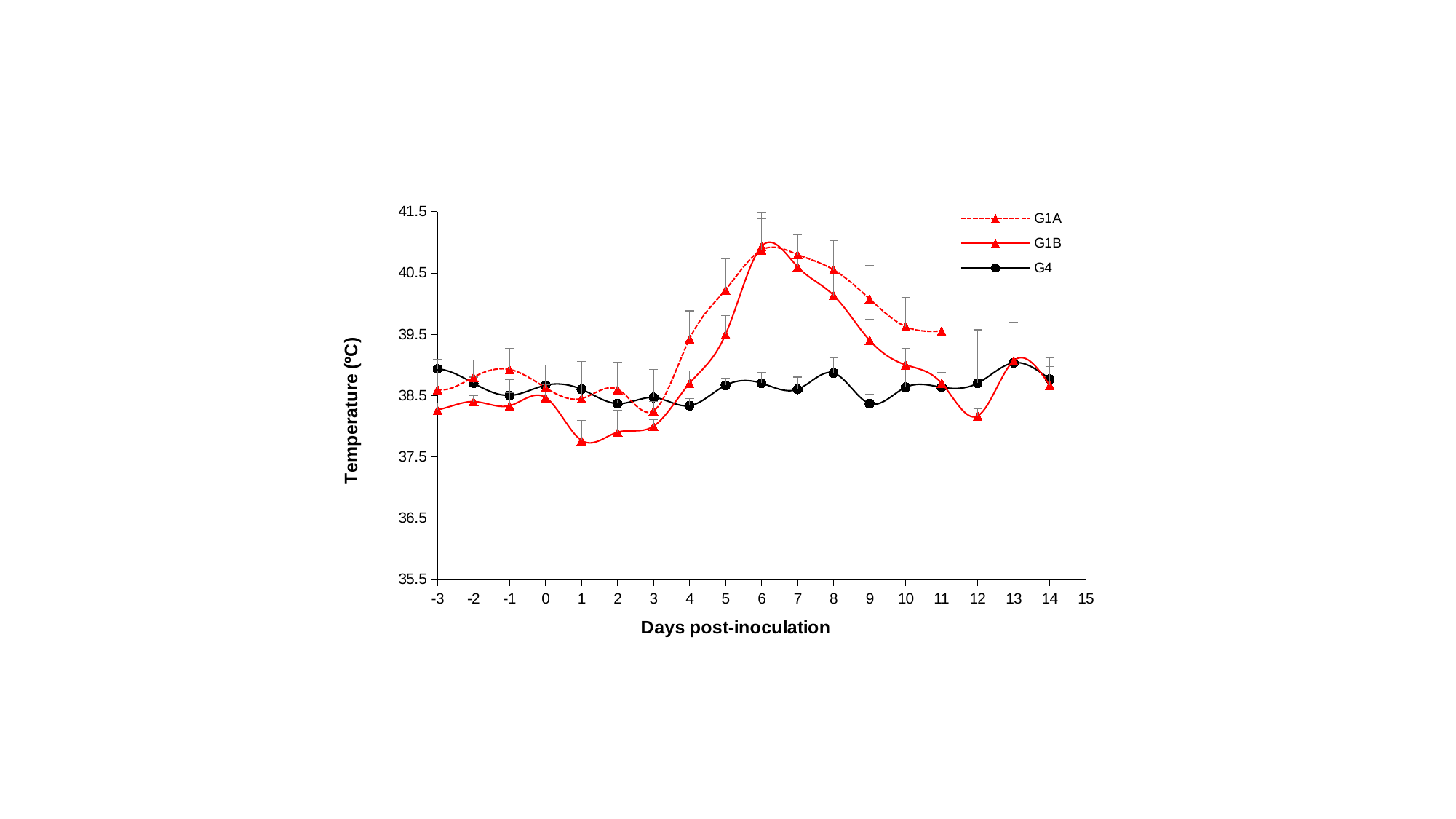

### Chart
| Category | G1A | G1B | G4 |
|---|---|---|---|

Supplement: Supplementary file 2 — 10.1186/s13567-016-0312-6 Mean rectal temperatures in G1. Mean rectal temperatures (+SD) recorded from goats inoculated with 106 Nc-Spain7 tachyzoites at day 40 of gestation that suffered foetal death during the second wpi (G1A) or during the third wpi (G1B) (see legend). [file 13567_2016_312_MOESM2_ESM.pptx]
